# A Female Hair Clip and Orthodontists' Neck-Back Pain Perception: A Survey

**DOI:** 10.1155/2021/6644470

**Published:** 2021-03-04

**Authors:** Arkan Muslim Al Azzawi, Hasan Sabah Hasan, Mohammed Nahidh, Mohamed Elkolaly, Ayshan Kolemen

**Affiliations:** ^1^Orthodontics Department, College of Dentistry-University of Babylon, Babil, Iraq; ^2^Orthodontic Department, Khanzad Teaching Center, General Directorate of Hawler-Ministry of Health, Erbil, Iraq; ^3^Orthodontics Department, College of Dentistry-University of Baghdad, Baghdad, Iraq; ^4^Orthodontic Department, Royal Dental Center, Alexandria, Egypt; ^5^Orthodontic Department, Al-Mustaqbal University College, Babel, Iraq

## Abstract

This study aimed to clarify the effect of large hair clips on patient head posture on the dental chair headrest and its harmful impact on orthodontist body posture and neck-back pain. One hundred orthodontists voluntarily participated in a web-based questionnaire designed and distributed online by using the Google form posted in the Telegram group of Iraqi orthodontists to assess the opinions of orthodontists regarding the effect of a large hair clip on the patient's position on the dental chair and site of pain perception during different stages of orthodontic treatment. Ninety percent of the orthodontists get bothered by the large hair clip. About 92% of the responses preferred their patients to remove the large hair clip; 99% of them responded that the large hair clip does affect the position of the patient's head on the chair's headrest. Eighty-nine percent responded that a large hair clip could disturb the operator during taking intraoral photographs, and 64% disturbed while taking dental impressions. Orthodontists reported that 4% had “back pain,” 28% had “neck pain,” and 60% had both “back and neck pain” during bonding appointment, while only 8% reported “no pain.” Regarding the activation appointments, 4% had “back pain,” 26% had “neck pain,” and 48% had both, while only 22% reported “no pain.” During the debonding appointments, 7% of the respondents had “back pain,” 29% had “neck pain,” and 44% had both “back and neck pain,” yet 20% stated absence of pain. Wearing a hair clip and changing patient position on dental chair and orthodontist posture during different stages of orthodontic work such as bonding, regular recall, and depending on the procedure may be directly related to the neck-back pain perception to an orthodontist.

## 1. Introduction

In ancient Greek, “Ergo” means work and “Nomos” means natural laws or systems. Thus, ergonomic is a science deals with designing and procedures for optimal efficiency and safety [1]. Additionally, it is a study of the correlation among the personnel, equipment, and environment in the work area [2]. Proper ergonomic design is necessary to prevent repetitive strain injuries that can develop over time and lead to long-term disability [3].

Work-related musculoskeletal disorders (WMSDs) are significant problems in modern societies [4, 5]. They refer to every kind of tissue damage to the musculoskeletal system and the nerves. Dentists are more likely to have musculoskeletal disorders due to their job that is highly dependent on patient position on the dental chair. Dentistry, especially general dentistry, is one of the stressful professions. Improper prolonged repetitive working habits and observational requirements of this field and recurrent movements of the upper body/limb are the reasons for these kinds of problems in this profession [6].

The determination of WMSD prevalence among orthodontists will provide researchers and practitioners with a relevant description of prevalence necessary to have an in-depth review of risk factors and ergonomic interventions. The average orthodontist suffers from work-related musculoskeletal disorder ranging from 45% up to 65%, which represented an alarming percentage to be considered by an orthodontist [7–12].

During recent years, dentist work environments have been studied in different parts of the world. Some researchers are convinced that the frequency of disorders has increased by changing standing and sitting positions. These researchers stated that this change of place while working might not be the only influential factor; other factors such as a work environment, mental pressure, nonstopping work periods, and dentists' visual status, which are highly dependent on direct or indirect access to patient mouth, are significant factors responsible for WMSDs [13].

Prolonged static postures are inherent in dentistry. Serious detrimental physiological changes in the body can result from these abnormal postures, including muscle imbalances, muscle necrosis, trigger points, hypomobile joints, nerve compression, spinal disk herniation, or degeneration. These changes often result in pain, injury, or MSDs. Preventing chronic pain in dentistry may require a paradigm shift within the profession regarding clinical work habits, including proper use of ergonomic equipment, frequent short stretch breaks, and regular strengthening exercise [14].

Hair clip is one of the traditional scarves used widely by females for tying hair; there are many types such as single and double, and small and large. The authors believed the presence of a relation between the large hair clip and the ergonomic of orthodontists and its consequences, so this study was conducted for the first time to establish this proposed relationship.

## 2. Material and Methods

Present cross-sectional web-based questionnaire study was conducted among the voluntary Iraqi orthodontists with at least 3-year experience; this is to establish up to 15 patients each day to be statistically significant when getting the orthodontist answer to survey. The research department-approved study protocol and design in the ministry of health due to the ministry of health supervised all governmental and private work. The questionnaire included the demographic information for all participants such as name, age, gender, residency, and year of experiencing orthodontics.

Questions prepared for this study were checked for validity and reliability by well-experienced orthodontists and statisticians who examined the questions to cover all aspects of the issue.

The questionnaire was designed to know the orthodontist's opinion regarding the effect of hair clips on the patient's position on the dental chair and detect the prevalence and site of pain perception during different orthodontic treatment stages shown in Tables 1 and 2.

The data collected using the Google form posted in the Telegram channel of Iraqi Orthodontists contained about 250 specialist orthodontists.

Data were collected and analyzed by the SPSS program (version 25). Frequency distribution and percentage were tabulated according to the gender of the participants, and differences were detected using Pearson's chi-square test with a 0.05 probability level.

## 3. Results

One hundred orthodontists (50 males and 50 females) with age averaged 37.79 ± 6.09 years participated in this questionnaire from different Iraqi cities with a 40% response rate.

Regarding the participants' replies to the first question, 90% of the participants get bothered by female patients wearing large hair clips; 94% were female orthodontists, and 86% were male orthodontists with no significant gender difference.

About 92% of the orthodontist preferred their patients to remove the large hair clip before starting the treatment. Female orthodontists chose to remove the hair clip more than males (94% and 90% respectively), with no significant gender difference.

For the question “whether the large hair clip affects the position of the patient's head on the chair's headrest,” 99% of the participants responded positively, amongst which 100% of males and 98% of females responded yes with no significant gender difference.

For the question about “whether a large hair clip does change the patient's position on the dental chair,” overall, 95% responded by yes; among them, 98% were females, and 92% were males, also with no significant gender difference.

Eighty-nine percent of the participants responded positively to “whether the large hair clip can disturb the operator during taking intraoral photographs,” and amongst them, there were 94% of females and 84% of males with no significant gender difference.

About 64% of the respondents (68% females and 60% males) were disturbed by the large hair clip while taking dental impressions, and again, no significant gender difference was reported, as shown in Table 1.

Regarding pain perception and location of pain during buccal bonding of fixed appliance appointments, 4% had back pain, 28% had neck pain, and 60% had back and neck pain; on the other hand, only 8% reported no pain. Comparing the results between males and females indicated a nonsignificant gender difference.

Regarding the pain during activation appointments, 4% had back pain, 26% had neck pain, and 48% had both back and neck pain, and only 22% did not report any pain with a significant gender difference (*p*=0.034).

During the debonding appointments, 7% of the respondents had back pain, 29% had neck pain, and 44% had both of them, yet 20% responded by absence of pain; again, the gender difference was significant (*p*=0.017), as shown in Table 2.

## 4. Discussion

The present study focused on observing the relationship of orthodontist neck and back pain with female patients wearing large hair clips. Most females in Islamic countries nowadays arrange their hairs with a large hair clip which is situated in the middle of the occipital bone facing the headrest of the dental chair.

To the best of the authors' knowledge, this is the first study conducted to address the effect of large hair clips on the pain perception among Iraqi orthodontists. The response rate for participation in this survey was 40% which was considered low.

Among all orthodontists participated in the questionnaire, 90% get bothered from female patients to arrange their hair with large hair clip because the majority of Iraqi are Muslims and the trend now is arranging the hair with large clip and this may change the direction of vision or change the setting of dental chair to an uncomfortable position.

Furthermore, the vast majority of orthodontists (92%) preferred their patients to remove the large hair clip before commencing the orthodontic visits; this may be attributed to the position of hair clip that facing the headrest of the dental chair, which may lead an orthodontist to change his/her visual direction.

Female orthodontists preferred the removal of hair clips before starting orthodontic treatment more than males (94% and 90%, respectively) with no significant gender difference. This preference in answering the questionnaire is related to hair clip and scarf because from a religious point of view, females can see the hair of other females, but this is prohibited to males.

**Figure 1:**
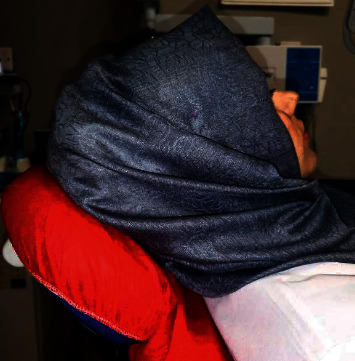
Head position on dental chair of a female patient wearing large hair clip underneath the scarf.

Additionally, almost all orthodontists, whether they are males or females, agreed that wearing of large hair clip would affect the position of the patient's head on the chair's headrest; this is maybe due to the position of the hair clip in the middle of the occipital bone that is directed to headrest of dental chair as shown in Figure 1.

About 89% and 64% of orthodontists claimed that large hair clip would disturb them during taking intraoral photographs and dental impression, respectively; this is maybe due to a close correlation between wearing a large hair clip and patient head position on a dental chair that will be tilted downward making buccal corridor area and peripheral seal area not accessible.

Dental professionals exhibit an elevated risk of acquiring musculoskeletal disorders (MSDs) than other professions because of the prolonged maintenance of static postures and precise hand and wrist movements. Regarding pain perception, 60% had back and neck pain. In comparison, only 8% reported no pain.

Regarding the pain during activation appointments, 4% had back pain, 26% had neck pain, and 48% had both back and neck pain, while only 22% reported no pain. During the debonding appointments, 7% of the orthodontists had back pain, 29% had neck pain, and 44% had both of them, yet 20% responded absence of pain, this is maybe the continuous movement of neck and shoulder during bonding, activation, and debonding in bad patient posture, this comes following the recommendation published by the National Institute for Occupational Safety and Health [15].

Furthermore, all Iraqi orthodontists utilized direct buccal bonding. No one used lingual appliances, so they spent more time and consequently caused back-neck pains in 60% of participants against 28% who suffered from neck pain. Generally, the direct bonding technique needed more attention and concentration to be done correctly; this requires more comfortable positioning of the patients' head on the dental chair headrest. Additionally, such long appointments may affect the neck of the patients too.

The more bonding sessions performed in the day, the higher possibility of experiencing pain. Research studies proved that one of the possible causes for work-related pain among dentists could be the imbalance in muscles between the lower back and abdominal muscles in the dental profession's sitting posture. Repeated leaning towards the patient may lead to strain and overexertion in the lower back extensor muscles. At the same time, the deep abdominal muscles of stabilization become weaker. Studies show that if the transverse abdominis muscle is healthy, the back pain level will decrease [16].

The appointments of fixed appliance activation entailed changing the elastomeric chains, activating the closing loops or NiTi retraction spring. This relatively short appointment may not hurt the orthodontists unless there are deboned brackets or loose bands. About 48% of the orthodontists suffered from both back and neck pains, while 26% reported neck pain, against 22% who reported no pain. Females experienced more neck pain, which might be related to the higher threshold of males' tolerance than females [17, 18].

The last appointment in the orthodontic treatment stages is debonding of orthodontic brackets. This takes a short time, but a longer time is needed to remove composite remnant on the tooth surface with scaling and polishing. Like the previous stage, pain in the back and neck was more prominent (44%), while 29% of the respondents reported neck pain only, while 20% declared no pain.

All of the participated orthodontists appreciated the current observational study. They suggested that female orthodontic patients should be instructed to remove the large hair clip or use a small one, if mandatory, before the appointment to avoid improper body position, leading to neck and back pain.

Orthodontists should keep the long axis of their torso vertical. The head should be tilted no more than 45° to the horizontal plane. Some corrective exercises should be executed every day may help prevent back pain. It is advisable for orthodontists to frequently change their position to avoid circulatory sluggishness and stiffness and muscle tightness. Moreover, stool with a comfortable padded seat, large enough to encompass the operator's buttock coverage, and two-thirds of the thigh should be selected to decrease the sitting position discomfort [19, 20].

This observational study's limitation was the low sample size (low response participation rate), limited number of questions in the questionnaire, and lack of correlation between various types of hair clips and therewith orthodontist performance and postural health.

Henceforth, a larger sample size with a global geographical distribution would be required. Although the survey was without any identification markers and respondents were assured of the results' confidentiality still, social desirability bias is the study's limitation. Another limitation is that it solely depends on the respondent's compliance with answering the questions honestly.

## 5. Conclusions

Within the limitations of the present study, it can be concluded that wearing a hair clip and changing patient position on dental chair and orthodontist posture during different stages of orthodontic work like bonding, regular recall, and depending on the procedure may be directly related to the neck-back pain perception to an orthodontist. The results of the current study are based on a low sample size. Nevertheless, it can provide a preliminary basis for extensive long-term sample-sized global studies.

## Figures and Tables

**Table 1:** Orthodontists' responses to the online questionnaire with gender difference.

Questions	Gender	Yes		No		Comparison
		*N*	%	*N*	%	
When you see a patient for the first time, do you get bothered by the large hair clip?	Female	47	94	3	6	*X* ^2^ = 1.778 d.f. = 1 *p* value = 0.182
	Male	43	86	7	14	
	Total	90	90	10	10	

When you see a patient with a large hair clip, do you prefer the patient to remove it?	Female	47	94	3	6	*X* ^2^ = 0.136 d.f. = *p* value = 0.712
	Male	45	90	5	10	
	Total	92	92	8	8	

Does the large hair clip affect the patient's head on the chair's headrest?	Female	49	98	1	2	*X* ^2^ = 0 d.f. = 1 *p* value = 1
	Male	50	100	0	0	
	Total	99	99	1	1	

Does the large hair clip change the position of the patient on the chair?	Female	49	98	1	2	*X* ^2^ = 0.842 d.f. = 1 *p* value = 0.359
	Male	46	92	4	8	
	Total	95	95	5	5	

Does the large hair clip disturb you while taking intraoral photographs?	Female	47	94	3	6	*X* ^2^ = 2.554 d.f. = 1 *p* value = 0.110
	Male	42	84	8	16	
	Total	89	89	11	11	

Does the large hair clip disturb you while taking dental impressions?	Female	34	68	16	32	*X* ^2^ = 0.694 d.f. = 1 *p* value = 0.405
	Male	30	60	20	40	
	Total	64	64	36	36	

**Table 2:** Orthodontists' responses to the online questionnaire regarding location of pain with gender difference.

Questions	Gender	Back pain		Neck pain		Both		None		Comparison
		*N*	%	*N*	%	*N*	%	*N*	%	
During the bonding appointment, what does the large hair clip cause?	Female	2	4	17	34	29	58	2	4	*X* ^2^ = 3.455 d.f. = 3 *p* value = 0.327
	Male	2	4	11	22	31	62	6	12	
	Total	4	4	28	28	60	60	8	8	
During fixed appliance activation visits, what does the large hair clip cause?	Female	2	4	18	36	24	48	6	12	*X* ^2^ = 8.664 d.f. = 3 *p* value = 0.034
	Male	2	4	8	16	24	48	16	32	
	Total	4	4	26	26	48	48	22	22	
During debonding appointments, what does the large hair clip cause?	Female	3	6	17	34	26	52	4	8	*X* ^2^ = 10.182 d.f. = 3 *p* value = 0.017
	Male	4	8	12	24	18	36	16	32	
	Total	7	7	29	29	44	44	20	20	

## Data Availability

The data that support the findings of this study are available from the corresponding author upon reasonable request.
